# Cytoprotective functions of amyloid precursor protein family members in stress signaling and aging

**DOI:** 10.1186/1750-1326-8-S1-P26

**Published:** 2013-09-13

**Authors:** Arpita Kundu, Andreas Zymny, Steffi Chang, Nelli Röhner, Donat Kögel

**Affiliations:** 1 Experimental Neurosurgery, Neuroscience Center, Goethe University Hospital, Frankfurt am Main, Germany

## Background

The amyloid precursor protein (APP) is processed via two different metabolic pathways: the amyloidogenic and the non-amyloidogenic pathway, the latter of which leading to generation of the secreted N-terminal APP fragment sAPPα [1]. Previous studies from our group suggest that sAPPα exerts potent neuroprotective effects and inhibits stress-triggered cell death via modulation of gene expression, as well as by antagonizing different types of neurotoxic stress [2]. It was also observed that the biochemical processing of APP is downregulated during aging which in turn reduced the secretion of sAPPα [3]. Based on these observations, we have studied the potential physiological function of sAPPα/APP and APLPs (APP like proteins) on the regulation of age-associated, stress induced signaling pathways, apoptosis and senescence.

## Materials and methods

SH-SY5Y, PC12, IMR90 cells were used as cellular models. Depletion of APP, APLP1 (APP like protein 1) and APLP2 (APP like protein 2) in SH-SY5Y cells was achieved by stable lentiviral knockdown. To analyze the protective function of sAPPα, we have used conditioned supernatants of wild type APP overexpressing HEK cells and recombinant His-tagged sAPPα purified from yeast. The cells were treated with sAPPα prior to the addition of different stress stimuli (MG132, epoxomicin, UV, H_2_O_2_) after which cell death, gene expression and senescence were analyzed by MTT assays, caspase activity assays, Western blots and X-Gal staining respectively.

## Results

Our data show that sAPPα can antagonize premature senescence induced by repetitive short term induction of proteasomal stress in IMR-90 cells and apoptosis triggered by prolonged proteasomal stress and other death stimuli in PC12, SH-SY5Y and IMR90 cells which was accompanied by a sAPPα-dependent inhibition of the JNK stress signaling pathway. In contrast, no significant changes in cell viability and apoptosis were observed when APP knockdown cells were pretreated with sAPPα.

## Conclusions

Our observations suggest that sAPPα can antagonize both apoptosis and cellular senescence and requires expression of holo-APP to mediate its cytoprotective effects. They also support the notion that the physiological function of APP is linked to modulation of neuronal and brain aging.

## References

[B1] Selkoe DJ Cell biology of protein misfolding: the examples of Alzheimer’s and Parkinson’s diseases Nat Cell Bio 2004 6 1054 1061 10.1038/ncb1104-1054 15516999

[B2] Kögel D Deller T Behl C Roles of amyloid precursor protein family members in neuroprotection, stress signaling and ageing Exp Brain Res 2012 217 471 479 10.1007/s00221-011-2932-4 22086493

[B3] Kern A Roempp B Prager K Walter J Behl C Down-regulation of endogenous amyloid precursor protein processing due to cellular aging J Biol Chem 2006 281 2405 2413 16303768 10.1074/jbc.M505625200

